# Empty pelvis syndrome as a cause of major morbidity after pelvic exenteration: validation of a core data set

**DOI:** 10.1093/bjs/znaf070

**Published:** 2025-04-30

**Authors:** Charles T West, Abhinav Tiwari, Julian Smith, Hideaki Yano, Malcolm A West, Alex H Mirnezami, Southampton Complex Cancer and Exenteration Team, G Ansell, A Bateman, C Birch, L Borthwick, H Cheema, V Dawson, K Donovan, J Douglas, R Exton, B George, J Green, M Hayes, G Hodges, L Ingram, C Lane, R Lewis, T Nash, M Nicolaou, B Patterson, E Ryan, Y Salem, D Spencer, K Stoddard, P Tapley, L Wodd, R Zaher

**Affiliations:** Southampton Complex Cancer and Exenteration Team, University Hospital Southampton NHS Foundation Trust, Southampton, UK; Academic Surgery, Cancer Sciences, University of Southampton, Southampton, UK; Academic Surgery, Cancer Sciences, University of Southampton, Southampton, UK; Southampton Complex Cancer and Exenteration Team, University Hospital Southampton NHS Foundation Trust, Southampton, UK; Urology Department, University Hospital Southampton NHS Foundation Trust, Southampton, UK; Southampton Complex Cancer and Exenteration Team, University Hospital Southampton NHS Foundation Trust, Southampton, UK; Southampton Complex Cancer and Exenteration Team, University Hospital Southampton NHS Foundation Trust, Southampton, UK; Academic Surgery, Cancer Sciences, University of Southampton, Southampton, UK; NIHR Southampton Biomedical Research Centre, Perioperative Medicine and Critical Care Theme, University Hospital Southampton NHS Foundation Trust, Southampton, UK; Southampton Complex Cancer and Exenteration Team, University Hospital Southampton NHS Foundation Trust, Southampton, UK; Academic Surgery, Cancer Sciences, University of Southampton, Southampton, UK

## Abstract

**Background:**

Pelvic exenteration (PE) is a potentially curative treatment for advanced pelvic cancers. However, PE procedures are associated with empty pelvis syndrome (EPS), a spectrum of complications including pelvic sepsis, sinus formation, fistulae, and bowel obstruction. Inconsistent reporting has impeded progress in understanding EPS. The PelvEx Collaborative introduced a core data set of descriptors and outcomes to address these issues and the aim of this study was to validate this data set.

**Methods:**

An observational cohort study applied the EPS core data set to a prospectively maintained PE database. Patterns of major and minor manifestations were evaluated; logistic regression was used to explore relationships between descriptors and outcomes, and inter-descriptor correlation was assessed using Cramer’s V.

**Results:**

EPS occurred in 32.1% of patients (105 of 327) and was the leading cause of major morbidity. Infected pelvic collections (occurring in 23.5%) were associated with subsequent chronic sinus formation (OR 3.08, *P* = 0.01) and fistulae (*P* = 0.05). The risk of EPS increased with external beam radiotherapy (OR 1.01 per 1 Gy, *P* = 0.01), sacrectomy (OR 3.78, *P* < 0.001), total cystectomy (OR 2.46, *P* = 0.001), internal iliac vessel ligation (unilateral OR 1.94, *P* = 0.045; bilateral OR 3.65, *P* < 0.001), and infralevator exenteration (OR 3.69, *P* < 0.001). Omentoplasty reduced pelvic bowel obstruction (OR 0.27, *P* = 0.004) and perineal flaps were linked to a higher rate of reconstruction-related major morbidity compared with biological mesh alone (20.8% *versus* 1.2% respectively, *P* = 0.002).

**Conclusion:**

The PelvEx Collaborative core data set standardizes reporting of EPS, with this study detailing the acute and chronic complications arising as a consequence. Biological mesh was associated with reduced reconstruction-related morbidity compared with perineal flaps. Further validation in additional cohorts is required to address potential confounding factors.

## Teaser

Empty pelvis syndrome is identified as the single most frequent cause of major morbidity after pelvic exenteration. A syndrome pattern in the natural history of these complications is observed. Omentoplasty is confirmed as being a useful technique to mitigate these complications and biological meshes appear to significantly reduce exposure to reconstruction-related morbidity.

## Introduction

In 1962, Brunschwig highlighted the importance of pelvic filling and the catastrophic consequences of an empty pelvis after pelvic exenteration (PE)^1,2^. PE has since become the standard of care for advanced pelvic cancers, but increasingly radical surgeries are increasing the risk of empty pelvis syndrome (EPS) complications^3,4^. Inconsistent reporting of EPS has led to fragmented research that has proven challenging to synthesize, prompting the PelvEx Collaborative to publish a first consensus definition, ‘EPS encompasses a spectrum of post-exenteration complications including infected fluid collections, bowel obstruction, perineal sinus, and fistulae—severity is multifactorial, likely due to radicality of resection and migration of bowel into the void generated’^5–7^. Interest in standardization of reporting in surgical studies has increased, leading to the development of core outcome sets that define critical consequences of a disease. More recently, core descriptor sets that stipulate key variables to describe patients with a particular pathology have also been developed^8,9^. To facilitate future research, the PelvEx Collaborative, alongside patient representatives, converted a longlist of 70 statements into a measurable core data set for EPS, with seven core outcomes and four core descriptors, summarized in *Table 1*.

**Table 1:** Measurable EPS core data set, as per the PelvEx Collaborative consensus^7^

EPS core data set item	Measurement
**Core outcomes**	
Infected pelvic collection	Suspected on CT with a radiologist’s report with no time constraint, to include collections in the pelvis or neo-perineum, or those relating to urinary or enteric leakage; only then counted if collection actively treated with either antibiotics or drainage*
Pelvic bowel obstruction	Diagnosed on CT with a transition point within the pelvis with no time constraint
Chronic perineal sinus	Chronic fluid discharge through an unhealed perineal or visceral wound (for example rectal or vaginal stump) for ≥6 months after surgery
Enteroperineal fistula	Any connection between the bowel and perineal wound or pelvic viscera to drain through the perineum with no time constraint
Morbidity from reconstruction	Any morbidity relating to the reconstructive technique used, including flap donor-site complications or explanted biological meshes with no time constraint
Re-interventions for EPS	Clavien–Dindo classification to grade any EPS complication and reporting of which re-interventions were required
Health-related quality of life	No validated instrument, so excluded from this pilot
**Core descriptors**	
Radiotherapy-induced damage	Dosages of preoperative EBRT reported cumulatively, with IOERT reported separately, both in Gy
Magnitude of surgery	PE coded using the UKPEN lexicon, which classifies radicality of resection in the compartments of the pelvis as ordinal scales^10^
Methods of reconstruction	Detail on any strategy used to fill or reconstruct the pelvis
Changes in the volume of pelvic dead space	No validated instrument, so excluded from this pilot

*Radiologist reports were not always conclusive about whether pelvic collections were infected or not; in these circumstances, they were only counted if CT subsequently influenced a clinical decision to actively manage pelvic or perineal sepsis. EPS, empty pelvis syndrome; EBRT, external beam radiotherapy; IOERT, intraoperative electron radiotherapy; PE, pelvic exenteration; UKPEN, UK Pelvic Exenteration Network.

Despite progress in the field, health-related quality of life (HRQoL) and changes in the volume of pelvic dead space do not yet have validated measurement instruments^7,11^. Morbidity from reconstruction was included to provide further insight into the safest strategies to mitigate EPS, with the PelvEx Collaborative reaching consensus in supporting omentoplasty and bulky myocutaneous flaps for reconstruction. However, evidence for these remains largely based on single-centre observational studies^6,7^. Use of biological mesh has also been reported in small series of PE, often combined in a composite manner with myocutaneous flaps for very substantial perineal defects^12–14^.

The aim of this study was to validate the PelvEx Collaborative EPS core data set by testing the following hypotheses: that EPS is an important cause of morbidity after PE; that, as a ‘syndrome’, its defined manifestations occur both acutely and chronically; and that the core descriptors are appropriate.

## Methods

The prospectively maintained Southampton Complex Cancer and Exenteration Team (SCCET) database was evaluated (2010–2024), determining the sample size. PE was defined as resection of two or more pelvic organs or compartments (palliative-intent PE and historical PE before the formation of the SCCET were included). Exclusions were standard pelvic resections, abdominal-only exenterations, duplicate redo PE, PE within the false pelvis only, and patients with <6 months of follow-up (to allow evaluation of chronic perineal sinuses). To report the magnitude of surgery, the UK Pelvic Exenteration Network (UKPEN) lexicon was applied, as per the EPS core data set, assigning the highest pelvic compartmental scores initially retrospectively using operation notes, histopathology reports, and reviews of postoperative imaging. Historical pelvic operations were added to scores, but additional resections (E1–E5) were excluded from the analysis^10^. Patients were then more broadly classified as having undergone either conventional or high-complexity PE, as recently described^15^.

The core data set was collected as stipulated in *Table 1*; however, HRQoL and changes in volumes of pelvic dead space were excluded due to a lack of validated measurement instruments. Complications from cancer recurrence, such as progressive lesions resulting in bowel obstruction, were not classified as EPS outcomes. Pelvic bowel obstruction, enteroperineal fistula, chronic perineal sinus, and infected pelvic collections were reported individually as manifestations of EPS and patients with any one of these were defined as having EPS. Morbidity from reconstruction was reported separately and not considered as a manifestation of EPS. The SCCET offer at least yearly follow-up, with imaging from referring hospitals centrally accessible and referring institutions contacted to minimize loss to follow-up. Missing data were assumed to be random and pairwise deletion was used throughout the analysis. Median follow-up was from surgery to death or the last known clinical or radiological follow-up.

The primary outcome was the overall rate of major EPS complications (Clavien–Dindo grade ≥IIIa). Secondary outcomes included minor EPS complications (Clavien–Dindo grade ≤II), re-intervention rate, time to onset, correlation between core descriptors and manifestation of EPS, EPS manifestation intercorrelation, and core descriptor intercorrelation.

All analyses were performed in R studio using percentages, medians, interquartile ranges, Shapiro–Wilk tests, ORs, and Fisher’s tests. Univariable logistic regression assessed the relationship of core descriptors to EPS manifestations, with significant descriptors placed into a multivariable logistic regression model that was additionally adjusted for prior pelvic surgery and sex. Cramer’s V was used to construct a correlation matrix for core descriptors, with correlation defined as being ≥0.10^16^.

Ethical approval was granted by NHS North East—Newcastle and North 2 Research Ethics Committee (REC:22/NE/0032), the database was registered on ClinicalTrials.gov (NCT05219058) as work package 1 of the Reconstruction in Extended MArgin Cancer Surgery Study with an analysis plan. A STROBE checklist is available in the *supplementary methods*^17^.

## Results

Of the 394 patients in the SCCET database, 27 underwent abdominal exenterations, 16 underwent PE within the false pelvis only, 5 underwent redo PE, and 19 had <6 months of follow-up. Baseline characteristics and EPS core descriptors of the remaining 327 patients are summarized in *Table 2*. Omentoplasty was used for pelvic filling in 64.5% of patients (211 of 327), regardless of whether PE was supralevator or infralevator. Among the 37.3% of patients (122 of 327) who underwent infralevator PE, perineal reconstruction was performed with biological mesh in 66.4% (81 of 122), myocutaneous flaps in 19.7% (24 of 122), composite reconstruction (biological mesh and flaps) in 12.3% (15 of 122), and primary closure only in 1.6% (2 of 122). Further details on reconstruction are in *Table S1*.

**Table 2:** Patient characteristics and EPS core descriptors; total number of patients = 327

Patient characteristics	Value
**Sex**	
Male	151 (46)
Female	176 (54)
**Age (years), median (interquartile range)**	63 (54–72)
**BMI (kg/m^2^), median (interquartile range)**	26 (24–30)
Smoking status*	
Current smoker	25 (8)
Ex-smoker	114 (35)
Non-smoker	186 (57)
**Co-morbidities**	
COPD/asthma	36 (11)
Diabetes	28 (9)
Heart disease	17 (5)
Chronic kidney disease	30 (9)
Hypertension	98 (30)
ASA grade*	
I	16 (5)
II	170 (57)
III	108 (36)
IV	3 (1)
**Diagnosis**	
Colorectal	219 (67)
Urological	59 (18)
Gynaecological	27 (8)
Anal	11 (3)
Other	11 (3)
**Cancer status**	
Primary disease	210 (64)
Recurrent disease	113 (35)
Benign disease	4 (1)
**Resection margin**	
R0	260 (80)
R1	50 (15)
R2	1 (0)
Palliative intent	9 (3)
Cytoreductive PE	3 (1)
Benign disease	4 (1)
**EPS core descriptors**	
Radiotherapy-induced damage	
Received pre-PE EBRT†	205 (63)
Received IOERT†	93 (30)
Magnitude of surgery	
Conventional supralevator PE	41 (13)
Conventional infralevator PE	29 (9)
High-complexity supralevator PE	164 (50)
High-complexity infralevator PE	93 (28)
Methods of reconstruction	
Pelvic filling	
Omentoplasty	211 (66)
Colorectal or coloanal anastomosis	17 (8)
Studer neobladder	2 (1)
Perineal reconstructions	
Biological mesh only	81 (66)
Myocutaneous flaps only	24 (20)
Composite (biological mesh + flaps)	15 (12)
Perineal primary closure only	2 (2)

Values are *n* (%) unless otherwise indicated. High-complexity and conventional PE are defined as per the UKPEN lexicon and recent studies^10,15^. Pelvic filling denotes techniques used to fill the pelvis. Omentoplasty was used regardless of the level of levator resection (327 patients), whereas colonic conduits or neobladders were used exclusively in supralevator PE (205 patients). Perineal reconstruction applies specifically to methods used in infralevator PE (122 patients). Composite reconstruction is defined as the use of both myocutaneous flaps and biological mesh for a single reconstruction. *Thirty historical ASA grades and two smoking statuses were missing. †Twenty patients enrolled in an IOERT double-blinded RCT and one missing dose of EBRT were treated as missing, as per the Methods. EPS, empty pelvis syndrome; COPD, chronic obstructive pulmonary disease; PE, pelvic exenteration; EBRT, external beam radiotherapy; IOERT, intraoperative electron radiotherapy; UKPEN, UK Pelvic Exenteration Network.

Between surgery and last follow-up, 28.1% of patients (92 of 327) experienced ≥1 major complication, with a total of 110 major complications overall. EPS manifestations were the most frequent cause of these (experienced by 11.9% of patients (39 of 327)). During the index admission, 65 distinct major complications occurred, with EPS again the leading cause (experienced by 7.3% of patients (24 of 327)). After discharge, there were 52 distinct major complications, with urological morbidity being the most common (experienced by 6.7% of patients (22 of 327)), followed by EPS (experienced by 6.4% of patients (21 of 327)). The median follow-up was 33 months. Additional detail on non-EPS complications is provided in *Table S2*.

EPS manifestations are summarized in *Table 3*. When including minor complications, EPS occurred in 32.1% of patients (105 of 327); 23.5% of patients (77 of 327) experienced an infected pelvic collection, 7.3% of patients (24 of 327) experienced a chronic perineal sinus, 7.0% of patients (23 of 327) experienced a pelvic bowel obstruction, and 0.6% of patients (2 of 237) experienced an enteroperineal fistula). EPS was diagnosed in 17.7% of patients (58 of 327) during the index admission and 14.4% of patients (47 of 327) after discharge.

**Table 3:** Manifestations of EPS, with detail as per the EPS core data set; total number of patients = 327

EPS manifestations	Value
**Any EPS complication**	105 (32)
Major	39 (37)
Minor	66 (63)
**Infected pelvic collection**	77 (24)
Major	31 (40)
Minor	46 (60)
Associated urinary leakage	1 (1)
Associated enteric leakage	3 (4)
Infected collection in the pelvis*	64 (83)
Infected collection in the neo-perineum	18 (23)
**Chronic perineal sinus**	24 (7)
Major	3 (13)
Minor	21 (88)
Empty pelvis sinus to perineal wound*	15 (63)
Empty pelvis sinus to vaginal stump	5 (21)
Empty pelvis sinus to penile urethral stump	2 (8)
Empty pelvis sinus to rectal stump	4 (17)
**Pelvic bowel obstruction**	23 (7)
Major	6 (26)
Minor	17 (74)
**Enteroperineal fistula**	2 (1)
Major	2 (100)
Minor	0 (0)
**Development of second EPS manifestation after diagnosis of infected pelvic collection, OR (95% c.i.), *P***	
Subsequent chronic perineal sinus	3.08 (1.29,7.30), 0.01
Subsequent pelvic bowel obstruction	1.85 (0.71,4.52), 0.20
Subsequent enteroperineal fistula	∞ (0.61,∞), 0.05

Values are *n* (%) unless otherwise indicated. Any EPS complication is defined as at least one of: infected pelvic collection, chronic perineal sinus, pelvic bowel obstruction, or enteroperineal fistula. Percentages for each individual complication are given under each bold subheading. *Patients could have co-synchronous collections/sinuses. EPS, empty pelvis syndrome.

The first diagnosed EPS manifestation was an infected pelvic collection in 22.9% of patients (75 of 327), a pelvic bowel obstruction in 4.9% of patients (16 of 327), and a chronic perineal sinus in 4.3% of patients (14 of 327); enteroperineal fistulae were always preceded by another EPS complication. An infected pelvic collection significantly increased the likelihood of subsequently developing a chronic perineal sinus (OR 3.08, *P* = 0.01) and showed a near-significant association with subsequent enteroperineal fistulation (OR ∞, *P* = 0.0549), but progression was not significant for pelvic bowel obstruction (OR 1.85, *P* = 0.20). Progression after other initial manifestations was not significant; however, one patient developed perineal wound dehiscence at 19 days that led to a chronic perineal sinus, followed by a delayed infected pelvic collection. Another patient presented initially with pelvic bowel obstruction at 2 months, followed by an infected pelvic collection and then an enterovaginal fistula. A total of 48 patients required unplanned readmission to manage EPS complications, of which 3.7% of patients (12 of 327) were readmitted to referring institutions and 11.0% of patients (36 of 327) were readmitted directly to the SCCET. A total of 5.2% of patients (17 of 327) required delayed re-intervention (>90 days) for EPS. Further detail on the timelines of presentations and management of individual EPS manifestations is provided in the *supplementary appendix*.

**Fig. 1:**
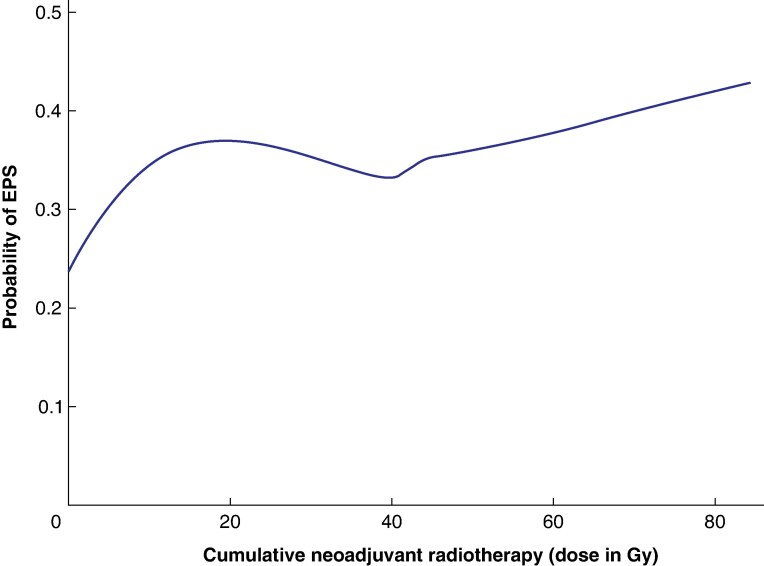
Locally estimated scatter plot smoothing line demonstrating the probability of developing an EPS complication with increasing doses of cumulative EBRT, *P* = 0.02 EPS, empty pelvis syndrome; EBRT, external beam radiotherapy.

Detail on univariable logistical regression analysis for all core descriptors against all EPS manifestations is shown in *Table S3*. Use of external beam radiotherapy (EBRT) was found to significantly increase the odds of developing EPS (OR 1.01 per 1 Gy, *P* = 0.02), but there was no significant association between intraoperative electron radiotherapy (IOERT) and the odds of developing EPS; these relationships are plotted in *Fig. 1* and *Fig. S1* respectively.

**Fig. 2:**
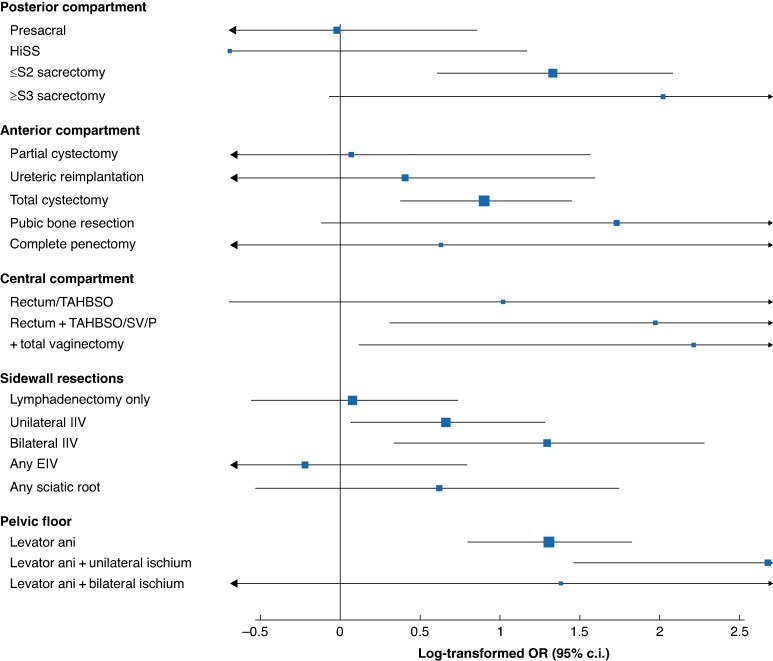
Forest plot summarizing logistical regression with log-transformed ORs for developing EPS complications based on increasing radicality in each pelvic compartment as defined by the UKPEN lexicon^10^ Coding for sidewall resections has been simplified to facilitate visualization; the level of IIV ligation is not considered and sciatic resections are grouped to be binary, combining all levels from single root to complete nerve resection (there were no bilateral sciatic resections). HiSS, high subcortical sacrectomy; TAHBSO, total abdominal hysterectomy with bilateral salpingo-oophrectomy; SV, seminal vesicles; P, prostate; IIV, internal iliac vessel; EIV, external iliac vessel; EPS, empty pelvis syndrome; UKPEN, UK Pelvic Exenteration Network. Produced in R and labelled in BioRender by C.T.W. (2025) https://BioRender.com/x02s125.

*Figure 2* demonstrates a trend for the escalating likelihood of EPS with increasing radicality in the posterior, anterior, central, lateral, and inferior compartments, compared with no resection in the corresponding compartment. This was significant for low sacrectomy (OR 3.78, *P* < 0.001), total cystectomy (OR 2.46, *P* = 0.001), unilateral internal iliac vessel ligation (OR 1.94, *P* = 0.045), bilateral internal iliac vessel ligation (OR 3.65, *P* = 0.008), infralevator PE (OR 3.69, *P* < 0.001), and infralevator PE with unilateral ischial spine resection (OR 14.58, *P* < 0.001). External iliac vessel resection (OR 0.80, *P* = 0.69) and sciatic nerve resection (OR 1.86, *P* = 0.28) did not have a significant influence.

Omentoplasty, performed in both supralevator and infralevator PE, significantly reduced the likelihood of pelvic bowel obstruction (OR 0.27, *P* = 0.004) compared with no omentoplasty, without any discrete omentoplasty-associated morbidity. Pelvic conduits did not significantly influence overall or individual EPS manifestations. Major morbidity directly relating to the perineal reconstruction itself was 1.2% (1 of 81) with the use of biological mesh and 20.8% (5 of 24) for myocutaneous flaps (*P* = 0.002).

On multivariable logistic regression, the level of complexity and the method of perineal reconstruction were independent predictors of EPS, whereas omentoplasty, cumulative EBRT dose, sex, and prior pelvic surgery were not (*Table S4*). The core descriptor intercorrelation matrix demonstrated 73.3% of comparisons (88 of 120) had at least moderate correlation (*Fig. S2*).

## Discussion

In this study, the first to utilize the PelvEx Collaborative core data set, EPS was the leading cause of overall and acute major morbidity, and the second highest cause of chronic major morbidity after PE. Previous studies report a similar prevalence, of 20.5–37.5%, with up to 59% of patients diagnosed late^18,19^.

Infected pelvic collections were the initial presentation in 71.4% and were associated with a significantly increased risk of chronic perineal sinus formation (OR 3.08, *P* = 0.01), implying that pelvic sepsis contributes to long-term perineal wound failure. A chronic perineal sinus was the first manifestation in 13.3% of patients, with one patient then developing a chronic pelvic collection, suggesting wound failure can lead to late contamination of an emptied pelvis.

Pelvic bowel obstruction was the initial presentation in 15.2% of patients, mostly diagnosed after discharge. Infected pelvic collections were not significantly associated with obstruction (*P* = 0.20), potentially due to the protective effect of omentoplasty (OR 0.27, *P* = 0.004). Low rates of bowel obstruction have also been reported with pelvic filling or exclusion with caecal mobilization, breast implants, obstetric balloons, or omentoplasty with mesh slings^19–22^. Conversely, in 2023, Nekkanti *et al*.^18^ did not utilize routine pelvic filling and described obstruction accounting for 45.3% of EPS. Therefore, mechanical incarceration of pelvic small bowel may be a distinct pathological step in the accumulation of infected fluid and subsequent development of EPS complications. Enteroperineal fistula is the most devastating EPS complication and the 0.6% rate reported in the present study compares favourably with the rates of 2.7–4.4% reported elsewhere^18,23^. This could be explained by the SCCET’s preference to exclude small bowel from a pelvis with cut ends of bone using biological mesh.

Preoperative EBRT is detrimental to perineal wound healing, but its cumulative effect on EPS has not been analysed previously^24^. For every 1 Gy administered, odds of EPS complications increased by 1% (*P* = 0.02). The highest cumulative dose was 85.6 Gy (45 Gy long-course EBRT, 30.6 Gy re-irradiation, and 10 Gy IOERT boost), reflecting the increasing dose escalation now possible in these patients. These small incremental increases were not significant in multivariable analysis and no association was observed with IOERT, perhaps due to the precise application of radiation minimizing wider pelvic damage^25^.

The trend of increasing EPS risk with increasing radicality across most pelvic compartments implies that increasing volumes of pelvic dead space and possible exposure of denuded bone are important in its development. Similarly, internal iliac vessel ligation significantly increased EPS, which may be due to resultant relative ischaemia in the lesser pelvis (facilitating propagation of pelvic sepsis) or the accompanying lymphadenectomy that invariably takes place with sidewall resections (increasing the potential for fluid accumulation).

**Fig. 3:**
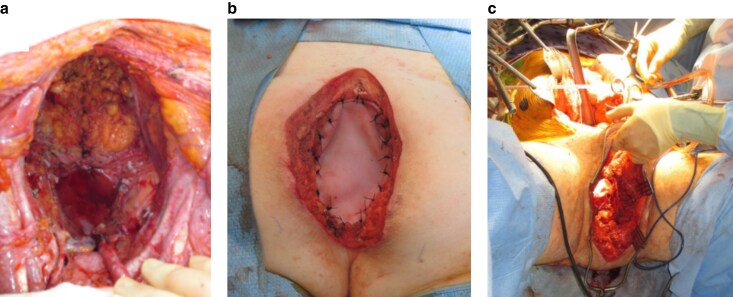
Pictorial demonstration of the heterogeneous nature of PE and the confounding that occurs when attempting to compare bespoke methods of reconstruction for patients having different magnitudes of surgery **a** Supralevator total PE. **b** Total infralevator PE with low sacrectomy reconstructed with a biological mesh. **c** Total infralevator PE with low sacrectomy and pubic bone resection requiring reconstruction with a flap in a patient who received more radiotherapy before surgery than the other examples. PE, pelvic exenteration.

Radiotherapy, magnitude of surgery, and relative pelvic ischaemia are usually a consequence of a radical approach to achieving R0 resection. The best approach to pelvic reconstruction after such radical surgery has been difficult to evaluate, with a suggestion that techniques should be tailored to individual patients^6,26^. Omentoplasty provides healthy, non-irradiated, and vascularized tissue for pelvic filling and, when combined with biological mesh, can promote neovascularization of these implants. The utility of omentoplasty demonstrated in this study has not been reported previously, but aligns with consensus from the PelvEx Collaborative^6,7^. This approach is not always reliable, as the omentum may be insufficient to reach, may be involved by tumour, or may be absent due to previous surgery, and, when present, may only fill 24.1% of dead space^27^. There was a significantly higher rate of major reconstruction-related morbidity when using myocutaneous flaps compared with biological mesh, which has been similarly noted in other series^19,28^. There is a preference at the SCCET for biological mesh with omentoplasty, restricting use of myocutaneous flaps to when the perineal skin defect is larger, for vaginal reconstruction, or after sacrectomy above the level of S4 (*Fig. 3*).

Overall, 73.3% of core descriptors exhibited at least moderate correlation in multivariable analysis, implying that the UKPEN lexicon has limitations by generating collinear confounders. It is currently unknown how its scales for different compartments interact, it does not account for patients that have undergone previous pelvic resections, and sex-specific classifications appear^10^. These issues were encountered in the multivariable model, as, when attempting to include the detail of pelvic compartmental resections of the UKPEN lexicon, convergence failure occurred due to unreliable parameter estimates that necessitated the complexity classification being used as a less detailed alternative^29^. Principal component analysis, propensity scoring, and larger stratified sample sizes could address the collinearity in the lexicon. This would allow both core descriptors, as well as other potentially relevant factors, such as age and sex, to be included in any model that would better align future study populations with those in randomized trials^15,30,31^.

This is an exploratory study in a single large-volume high-complexity PE unit in the UK, so has limited generalizability. Although historical PE were included, before mitigation of EPS was considered an essential part of PE, most had pelvic filling to reduce complications, representing a potential limitation that may explain the relatively low rates of serious EPS-related issues in this study. A volumetric EPS definition that reliably associates with complications could simplify analysis and enable the estimation of pelvic dead space from preoperative scans, allowing prediction of risk of EPS and proactive modification of pelvic filling strategies^27^. Additionally, it could be helpful to assess EPS complications in relation to postoperative imaging, where the dead space was inadequately filled or expands over time due to myocutaneous flap atrophy or perineal herniation.

With PE surgery becoming more radical, the high rates of EPS discussed seem likely to increase and, therefore, further focus on the optimal management of these complications is important. Sutton *et al*.^23^ found that 6% of patients needed surgical re-intervention for EPS, similar to the 5.2% of patients in this study. They identified controversies around resecting or bypassing obstructed or fistulating pelvic small bowel loops^23^. As detailed in the *supplementary appendix*, a resectional approach was used in the present study, with the two fistulae excised with good outcomes. However, de-incarceration of obstructed pelvic bowel loops resulted in one ureteric injury and one enterocutaneous fistula, both after emergency surgery in the patients’ local hospitals. Furthermore, of the 48 unplanned readmissions for EPS, 25% were to these local institutions and, therefore, wider awareness and a low threshold for involving experienced PE units are recommended.

The most pressing priority is understanding the impact of EPS on HRQoL, an agreed core outcome from the PelvEx Collaborative^7^. This study implies that myocutaneous flaps may increase exposure to reconstruction-related morbidity, which at times can be more severe than complications caused by EPS. Patient perceptions are needed to facilitate the balancing of these complex risks to recommend approaches resulting in the most favourable overall patient experiences. Ongoing parallel studies indicate EPS may contribute to regret after PE and can delay patient adaptation to their new post-PE normal^11,32^.

This study validates the EPS core data set, confirming consensus with significant data that highlights EPS as one of the most important causes of PE-related morbidity. A syndrome pattern is seen, encompassing both acute and chronic manifestations. There are strong correlations to core descriptors, with the risk of EPS escalating with both radiotherapy and surgical radicality. Core outcome sets have faced limited adoption; however, the EPS core data set was designed to be internationally reproducible and has now been implemented successfully^33^. Additional cohorts will be able to confirm or refute the findings of the present study, providing a scaffold for future multicentre comparative work that will be able to evaluate different reconstructive modalities designed to avoid EPS. Historically, PE surgery has prioritized oncological outcomes; however, with improving survival, there is a shift towards improving the quality of this extended survivorship and reducing morbidity from EPS remains a key part of this.

## Collaborators

G. Ansell; A. Bateman; C. Birch; L. Borthwick; H. Cheema; V. Dawson; K. Donovan; J. Douglas; R. Exton; B. George; J. Green; M. Hayes; G. Hodges; L. Ingram; C. Lane; R. Lewis; T. Nash; M. Nicolaou; B. Patterson; E. Ryan; Y. Salem; D. Spencer; K. Stoddard; P. Tapley; L. Wodd; R. Zaher (Southampton Complex Cancer and Exenteration Team, Southampton, UK).

## Supplementary Material

znaf070_Supplementary_Data

## Data Availability

The data sets generated during and analysed during the present study are available from the corresponding author upon reasonable request.
