# Supplementary material for: Empty pelvis syndrome as a cause of major morbidity after pelvic exenteration: validation of a core data set
Source: Br J Surg. 2025 Apr 30;112(5):znaf070. doi: 10.1093/bjs/znaf070 (PMC12043005; doi:10.1093/bjs/znaf070)
Supplement: znaf070_Supplementary_Data [file znaf070_supplementary_data.docx]

**Title:** Empty pelvis syndrome as a cause of major morbidity after pelvic exenteration: Validation of a core dataset

**Authors**

CT West^1,2^ (Conceptualization, data curation, formal analysis, funding acquisition, investigation, methodology, project administration, visualization, writing – original draft)

A Tiwari^2^ (Investigation, writing – review & editing)

J Smith^1,3^ (Resources, writing – review & editing)

H Yano^1^ (Conceptualization, resources, writing – review & editing)

MA West^1,2,4*^ (Conceptualization, methodology, resources, supervision writing – review & editing)

AH Mirnezami^1,2*^ (Conceptualization, methodology, resources, supervision, validation, writing – review & editing)

On behalf of SCCET

**Affiliation:**

^1^Southampton Complex Cancer and Exenteration Team, University Hospital Southampton NHS Foundation Trust, Southampton, UK

^2^Academic Surgery, Cancer Sciences, University of Southampton, Southampton, UK

^3^Urology Department, University Hospital Southampton NHS Foundation Trust, Southampton, UK

^4^NIHR Southampton Biomedical Research Centre, Perioperative Medicine and Critical Care theme, University Hospitals Southampton NHS Foundation Trust, Southampton, UK

**Corresponding author.**

Professor Alex Mirnezami / Associate Professor Malcolm West

University Surgery,

Room AC67, Mail Point 816, Level C,

South Academic Block,

University Hospital Southampton NHS Foundation Trust,

Tremona Road,

Southampton,

SO16 6YD

[alex.mirnezami@uhs.nhs.uk](mailto:alex.mirnezami@uhs.nhs.uk) or [m.west@soton.ac.uk](mailto:m.west@soton.ac.uk)

**ORCID ID**; 0000-0002-0345-5346 **Twitter**; @drmalcolmwest

**Supplementary Materials - Index**

| **Supplementary Methods** |  |
| --- | --- |
| Supplementary File 1 (STROBE Statement) | *pag. 4 – 5* |
| **Supplementary Appendix** |  |
| Details on the timelines and specific management of empty pelvis syndrome | *pag. 6 – 7* |
| **Supplementary Figures and Tables** |  |
| Figure S1 – IOERT and empty pelvis syndrome plot | *pag. 8* |
| Figure S2 – Empty pelvis syndrome core descriptor correlation matrix | *pag. 9* |
| Table S1 – Detail on method and morbidity from reconstruction | *pag. 10 – 11* |
| Table S2 – Causes of overall, index admission, and follow-up major morbidity | *pag. 12 – 13* |
| Table S3 – Logistical regression for all manifestations of empty pelvis syndrome for all core descriptors | *pag. 14 – 18* |
| Table S4 - Multivariable logistic regression of all empty pelvis syndrome (EPS) core descriptors for any EPS manifestation | *pag. 19* |
| **References** | *As per main manuscript* |
|  |  |

**Supplementary Methods**

STROBE Statement—checklist of items that should be included in reports of observational studies.

|  | Item | Recommendation | Location within respective section of manuscript |
| --- | --- | --- | --- |
| **Title and abstract** | 1 | (*a*) Indicate the study’s design with a commonly used term in the title or the abstract | Abstract |
|  |  | (*b*) Provide in the abstract an informative and balanced summary of what was done and what was found | Abstract |
| Introduction | | | |
| Background/rationale | 2 | Explain the scientific background and rationale for the investigation being reported | Paragraphs 1, and 2 |
| Objectives | 3 | State specific objectives, including any prespecified hypotheses | Paragraph 3 |
| Materials and Methods | | | |
| Study design | 4 | Present key elements of study design early in the paper | Paragraph 1 |
| Setting | 5 | Describe the setting, locations, and relevant dates, including periods of recruitment, exposure, follow-up, and data collection | Paragraph 1 |
| Participants | 6 | (*a*) *Cohort study*—Give the eligibility criteria, and the sources and methods of selection of participants. Describe methods of follow-up | Paragraphs 1 and 2 |
|  |  | (*b*) *Cohort study*—For matched studies, give matching criteria and number of exposed and unexposed | N/A |
| Variables | 7 | Clearly define all outcomes, exposures, predictors, potential confounders, and effect modifiers. Give diagnostic criteria, if applicable | Table 1, paragraphs 2 and 3 |
| Data sources/ measurement | 8* | For each variable of interest, give sources of data and details of methods of assessment (measurement). Describe comparability of assessment methods if there is more than one group | Table 1, paragraph 2 |
| Bias | 9 | Describe any efforts to address potential sources of bias | Paragraphs 1, 2, and 4 |
| Study size | 10 | Explain how the study size was arrived at | Paragraph 1 |
| Quantitative variables | 11 | Explain how quantitative variables were handled in the analyses. If applicable, describe which groupings were chosen and why | Paragraph 5 |
| Statistical methods | 12 | (*a*) Describe all statistical methods, including those used to control for confounding | Paragraph 4 |
|  |  | (*b*) Describe any methods used to examine subgroups and interactions | Paragraphs 3 and 4 |
|  |  | (*c*) Explain how missing data were addressed | Paragraph 2 |
|  |  | (*d*) *Cohort study*—If applicable, explain how loss to follow-up was addressed | Paragraph 2 |
|  |  | (*e*) Describe any sensitivity analyses | N/A |
| **Results** | | | |
| Participants | 13* | (a) Report numbers of individuals at each stage of study—eg numbers potentially eligible, examined for eligibility, confirmed eligible, included in the study, completing follow-up, and analysed | Paragraph 1 |
|  |  | (b) Give reasons for non-participation at each stage | Paragraph 1 |
|  |  | (c) Consider use of a flow diagram | See graphical abstract |
| Descriptive data | 14* | (a) Give characteristics of study participants (eg demographic, clinical, social) and information on exposures and potential confounders | Paragraph 1, and Table 2 |
|  |  | (b) Indicate number of participants with missing data for each variable of interest | Table 2, and Table S4 |
|  |  | (c) *Cohort study*—Summarise follow-up time (eg, average and total amount) | Paragraph 2, and Table S2 |
| Outcome data | 15* | *Cohort study*—Report numbers of outcome events or summary measures over time | Paragraphs 2, 3, 4, 5, 6 and Appendix, Table 3, Table S2, Table S3, Table S4, Figure 1, Figure 2, Figure S1 |
| Main results | 16 | (*a*) Give unadjusted estimates and, if applicable, confounder-adjusted estimates and their precision (eg, 95% confidence interval). Make clear which confounders were adjusted for and why they were included | Paragraphs 8, Figure 2, Table 3, Table S3, and Table S4 |
|  |  | (*b*) Report category boundaries when continuous variables were categorized | Figure S2 |
|  |  | (*c*) If relevant, consider translating estimates of relative risk into absolute risk for a meaningful time period | NA |
| Other analyses | 17 | Report other analyses done—eg analyses of subgroups and interactions, and sensitivity analyses | Paragraph 7 and 8, Table S1, Figure S2 |
| **Discussion** | | | |
| Key results | 18 | Summarise key results with reference to study objectives | Paragraphs 1 and Conclusions |
| Limitations | 19 | Discuss limitations of the study, taking into account sources of potential bias or imprecision. Discuss both direction and magnitude of any potential bias | Paragraphs 5 and 6 |
| Interpretation | 20 | Give a cautious overall interpretation of results considering objectives, limitations, multiplicity of analyses, results from similar studies, and other relevant evidence | Throughout discussion |
| Generalisability | 21 | Discuss the generalisability (external validity) of the study results | Paragraph 5 |
| **Other information** | | | |
| Funding | 22 | Give the source of funding and the role of the funders for the present study and, if applicable, for the original study on which the present article is based | Research funding statement |

*Give information separately for cases and controls in case-control studies and, if applicable, for exposed and unexposed groups in cohort and cross-sectional studies.

**Note:** Adapted from [www.strobe-statement.org](https://protect.checkpoint.com/v2/___http://www.strobe-statement.org___.bXQtcHJvZC1jcC1ldXcyLTE6dW5pdmVyc2l0eWhvc3BpdGFsc291dGhhbXB0b246YzpvOmMzMWE3YjFkMjRhNDczYTYwYTQyYjY1ODM0ZjRmNjgxOjY6ZTFhNDo4MDdjZDQxZjgwMzJkMzk4MjQ5NDEyMzY2MzVkMWUyODkzNTA3ZTE0YmI2NmYxMDljZDkzZTgzMDE3YzA1M2QxOnA6VDpO) with references to case-control and cross-sectional studies removed.

**Supplementary Appendix**

**Details on the timelines and specific management of empty pelvis syndrome:**

*Infected pelvic collections:*

Of the 77 infected pelvic collections 52 were diagnosed acutely during the index admission, and of these 55.8% (29/52) were managed conservatively, 28.8% (15/52) had single interventional radiological (IR) drainage, 9.6% (5/52) multiple IR drainages, and 5.8% (3/52) underwent surgical drainage. Following discharge 68.0% of infected pelvic collections (17/25) were treated conservatively, 12.0% (3/25) single IR drainage, 4.0% (1/25) multiple IR drainages, 4.0% (1/25) urethral catheter insertion, 12.0% (3/25) surgical drainage. 76.6% (59/77) were located in the pelvis, 16.9% (13/77) in the neo-perineum, and 6.5% (5/77) in both these regions above and below a perineal reconstruction. All neo-perineal collections occurred in infralevator cases, occurring in 14.8% (12/81) of biological meshes, 8.3% (2/24) of myocutaneous flaps, and 26.7% (4/15) of composite reconstructions.

*Chronic perineal sinuses:*

As per the core dataset, chronic perineal sinuses had to have been documented for at least 6 months following PE. Development of a chronic perineal sinus was significantly associated with infralevator PE (p<0.001). Of the 24 chronic perineal sinuses diagnosed, the first documented date of a non-healing wound or chronic discharge was a median of 57 days post-surgery (IQR 82). 21/24 (87.5%) of these were minor complications, with two cases undergoing examination under anaesthesia with curettage, and one a superior gluteal artery perforator flap repair due to exposed bone. 60.0% (15/25) of sinuses were confined to the perineal wound, while the remaining 40.0% (10/25) involved both visceral (e.g., rectal or vaginal stump) and/or perineal wounds.

*Pelvic bowel obstruction:*

Pelvic bowel obstruction was diagnosed in 23 patients (7.0%), and of these the majority (14/23, 60.9%) presented following discharge, at a median of 487 days (IQR 1,393) after PE. Four patients required re-laparotomy, with one perineal hernia repair, one small bowel resection, and two pelvic adhesiolyses. Two of these cases were performed at referring district general hospitals and sustained significant subsequent complications with one ureteric injury and one enterocutaneous fistula. All early pelvic bowel obstructions were managed conservatively.

*Enteroperineal fistulas:*

Two patients (<1%) developed enteroperineal fistulas in this series. These both had infected pelvic collections diagnosed at 84- and 384-days post-surgery, with fistulas subsequently identified at 106 and 1 047 days respectively. There was one enterovaginal fistula, and one enterocutaneous fistula in a supralevator case that eroded through the ischiorectal skin. These were managed in conjunction with the co-located intestinal failure team using control with drainage, nutritional optimisation with either home or inpatient parenteral nutrition, followed by delayed fistula resection, and myocutaneous flap reconstructions. Neither was associated with local or systemic recurrence of cancer.

**Supplementary Figures and Tables**

**
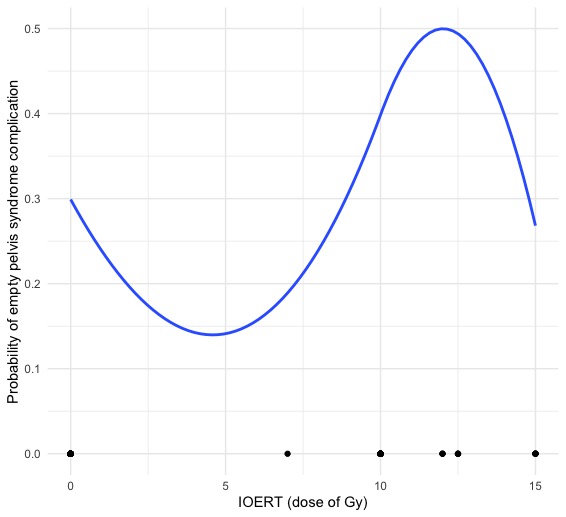
**

Figure S1 – Locally estimated scatterplot smoothing line demonstrating the probability of developing an empty pelvis syndrome complication with increasing doses of intraoperative electron radiotherapy (IOERT), p = 0.13.


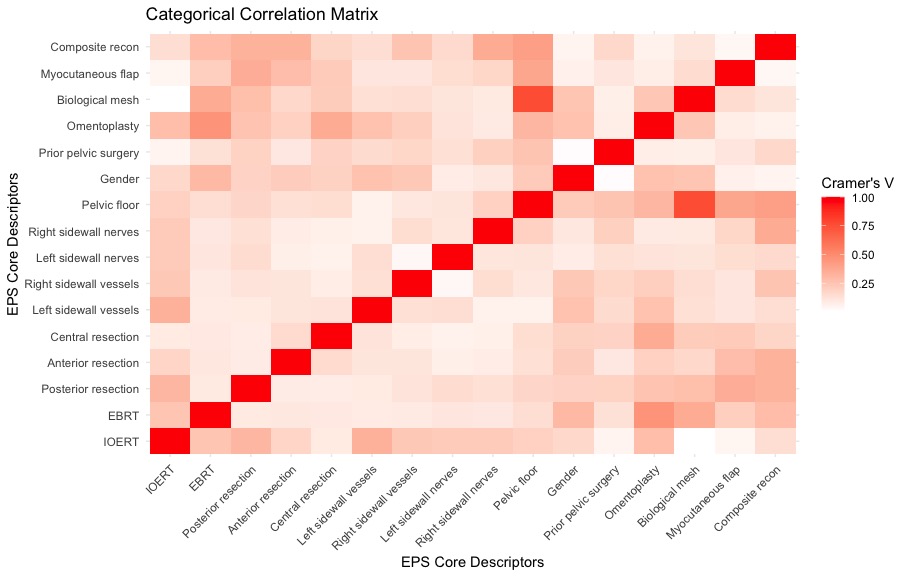


Figure S2 – Cramer’s V correlation matrix heatmap comparing all empty pelvis syndrome (EPS) core descriptors against each other. Note that intra-operative electron radiotherapy (IOERT) was converted to a binary variable, and external beam radiotherapy (EBRT) categorised by 0 Gy, 1 – 30.6 Gy, 31 – 50 Gy, 50.4 – 55 Gy, and >56 Gy.

| **Detail on method and morbidity from reconstruction** | | |
| --- | --- | --- |
| *Reconstruction method* | *n (%)* | *Morbidity from reconstruction* |
| **Omentoplasty** | 211 (65) | None |
| **Biological mesh** | 81 (68) | |
| SurgiMend 4.0 | 76 (94) | Partial explanation of one mesh involved in enteroperineal fistula |
| Ovitex PRS 2S | 1 (1) | None |
| Strattice extra thick | 3 (4) | None |
| Biodesign | 1 (1) | None |
| **Myocutaneous flap** | 24 (20) | |
| *Abdominal* | 20 (83) | |
| Vertical rectus abdominis muscle | 19 (79) | 1 donor site major dehiscence, 3 donor site local infections, 1 donor site fluid collection,  1 major and 7 minor flap dehiscences, 1 partial flap loss, 1 flap necrosis |
| Deep inferior epigastric perforator | 1 (4) | Nil |
| *Perineal* | 2 (8) | |
| Hatchet flap | 1 (4) | 1 minor flap dehiscence |
| Lotus petal | 1 (4) | 1 minor flap and donor site dehiscence |
| *Thigh* | 2 (8) | |
| Profunda artery perforator | 1 (4) | Nil |
| Gracilis | 1 (4) | 1 major flap dehiscence |
| **Composite reconstructions** | 15 (13) | |
| Strattice extra thick + Abdominal | 1 (7) | Nil |
| Strattice extra thick + Thigh | 1 (7) | Nil |
| SurgiMend 4.0 + Local | 2 (13) | 1 minor flap dehiscence |
| SurgiMend 4.0 + Thigh | 7 (47) | 1 flap necrosis, 4 minor flap dehiscences, 1 donor site major dehiscence |
| SurgiMend 4.0 + Gluteal | 1 (7) | 1 flap necrosis |
| SurgiMend 4.0 + Abdominal + Thigh | 3 (20) | 1 donor site major dehiscence, 1 superficial flap dehiscence |

Table S1 – Detail on the methods used for reconstruction with their associated reconstruction-related morbidity. Heterogeneity of myocutaneous flaps performed is due to plastic surgeon preferences, heterogeneity of the defect, and use of previous donor sites in cases of recurrence.

| **Major complications** | **n = 327** |
| --- | --- |
|  |  |
| **Overall major complication rate,** *n* (%)* | 92 (28) |
| *Total distinct major complications* | 110 |
| Empty pelvis syndrome | 39 (12) |
| Urological complications | 27 (8) |
| Cardiorespiratory complications | 11 (3) |
| Morbidity from reconstruction | 9 (3) |
| Abdominal complications | 7 (2) |
| Wound complications | 6 (2) |
| Long line complications | 6 (2) |
| Stoma complications | 4 (1) |
| Lymphoedema | 1 (0) |
| **Index admission major morbidity rate**, *n** *(%)* | 62 (19) |
| *Total distinct major complications* | 65 |
| Empty pelvis syndrome | 24 (7) |
| Cardiorespiratory complications | 11 (3) |
| Morbidity from reconstruction | 7 (2) |
| Long line complications | 6 (2) |
| Abdominal complications | 6 (2) |
| Wound complications | 5 (2) |
| Urological complications | 5 (2) |
| Stoma complications | 1 (0) |
| **Follow-up major morbidity rate**, *n** *(%)* | 49 (15) |
| *Total distinct major complications* | 52 |
| Urological complications | 22 (7) |
| Empty pelvis syndrome | 21 (6) |
| Morbidity from reconstruction | 3 (1) |
| Stoma complications | 3 (1) |
| Lymphoedema | 1 (0) |
| Wound complications | 1 (0) |
| Abdominal complications | 1 (0) |
| **Median follow-up**, *months (IQR)* | 33 (29) |

Table S2 – Causes of overall, index admission, and follow-up major morbidity. Individual patients may have had multiple major complications, therefore numbers of distinct complications are also given in each category. The overall complications are derived from collating index admission, and follow-up major morbidity, note that if the same complication required repeated intervention over the index admission and during follow-up then it was not counted twice.

IQR = interquartile range.

| **Univariate logistical regression of core descriptors** | **Any empty pelvis syndrome manifestation, OR (CI) p-value, n=105** | **Infected pelvic collection, OR (CI) p-value, n=77)** | **Chronic perineal sinus (n=24)** | **Pelvic bowel obstruction (n=23)** | **Enteroperineal fistula (n=2)** |
| --- | --- | --- | --- | --- | --- |
|  |  |  |  |  |  |
| **Radiotherapy induced damage (per 1 Gy increase in dose)** |  |  |  |  |  |
| Cumulative pre-PE EBRT dose (median = 45 Gy, IQR 50) | 1.01 (1.002 – 1.02); p = 0.02 | 1.009 (0.99 – 1.02); p = 0.11 | 1.02 (1.003 – 1.04); p = 0.03 | 0.99 (0.98 – 1.01); p = 0.55 | 1.09 (0.80 – 1.41); p = 0.49 |
| IOERT dose (median = 0 Gy, IQR 0) | 1.04 (0.99 – 1.09); -p = 0.13 | 1.04 (0.99 – 1.10); p = 0.10 | 1.06 (0.98 – 1.15); p = 0.14 | 0.96 (0.86 – 1.05); p = 0.49 | 1.03 (0.97 – 1.12); p = 0.42 |
| **Magnitude of surgery (compared to no resection in each compartment)** |  |  |  |  |  |
| *Posterior compartment* |  |  |  |  |  |
| No posterior component (n = 257) | - | - | - | - | - |
| Pre-sacral (n = 25) | 0.98 (0.37 – 2.35), p = 0.97 | 0.96 (0.31 – 2.51), p = 0.94 | 0.72 (0.04 – 3.85), p = 0.76 | 1.23 (0.19 – 4.55), p = 0.79 | 10.67 (0.41 – 275.52), p = 0.10 |
| High subcortical sacrectomy (n = 6) | 0.50 (0.03 – 3.20), p = 0.54 | 0.77 (0.04 – 4.91), p = 0.81 | 3.47 (0.17 – 23.55), p = 0.27 | 0.00 (NA), p = 1.00 | 0.00 (NA, p = 1.00 |
| Low sacrectomy (n = 35) | 3.78 (1.84 – 7.99), p <0.001 | 2.89 (1.37 – 6.00), p = 0.005 | 5.14 (1.91 – 13.18), p = 0.001 | 1.82 (0.50 – 5.31), p = 0.31 | 0.00 (NA), p = 1.00 |
| High sacrectomy (n = 4) | 7.56 (0.95 – 154.23), p = 0.082 | 11.55 (1.45 – 236.02), p = 0.036 | 0.00 (NA), p = 0.99 | 0.00 (NA), p = 1.00 | 0.00 (NA), p = 1.00 |
| Sacrectomy requiring stabilisation (n = 0) | - | - | - | - | - |
| *Anterior compartment* |  |  |  |  |  |
| No anterior component (n = 119) | - | - | - | - | - |
| Partial cystectomy (n = 9) | 1.07 (0.15 – 4.78), p = 0.93 | 0.87 (0.05 – 5.23), p = 0.90 | 0.00 (NA), p = 0.99 | 2.00 (0.10 – 13.42), p = 0.54 | 1.00 (NA), p = 1.00 |
| Ureteric resection with reimplantation (n = 14) | 1.50 (0.39 – 4.92), p = 0.52 | 2.77 (0.69-9.52), p = 0.12 | 0.00 (NA), p = 0.99 | 0.00 (NA), p = 0.99 | 1.00 (NA), p = 1.00 |
| Total cystectomy / radical cystoprostatectomy (n = 177) | 2.46 (1.46 – 4.25), p = 0.001 | 2.96 (1.61 – 5.73), p = 0.001 | 3.25 (1.18 – 11.5), p = 0.04 | 1.37 (0.55 – 3.73), p = 0.51 | >1000 (NA), p = 1.00 |
| Cystectomy with pubic bone resection (n = 5) | 5.64 (0.89 – 44.61), p = 0.66 | 10.4 (1.60 – 84.04), p = 0.014 | 19.17 (2.11 – 154.54), p = 0.005 | 0.00 (NA), p = 0.99 | 1.00 (NA), p = 1.00 |
| Cystectomy with complete penectomy (n = 3) | 1.88 (0.09 – 20.40), p = 0.61 | 3.47 (0.16 – 38.4), p = 0.32 | 0.00 (NA), p = 0.99 | 8.00 (0.35 – 94.32), p = 0.11 | 1.00 (NA), p = 1.00 |
| *Central compartment* |  |  |  |  |  |
| No central component (n = 12) | - | - | - | - | - |
| Rectum OR TAHBSO (n = 109) | 2.78 (0.50 – 52.19), p = 0.34 | 1.76 (0.31 – 33.24), p = 0.60 | >1000 (NA), p = 0.99 | >1000 (NA), p = 0.99 | 1.00 (NA), p = 1.00 |
| Rectum AND TAHBSO / partial vaginectomy  OR seminal vesicle / partial or complete prostatectomy (n = 195) | 7.18 (1.36 – 132.53), p = 0.062 | 4.66 (0.88 – 86.10), p = 0.15 | >1000 (NA), p = 0.99 | >1000 (NA), p = 0.99 | >1000 (NA), p = 1.00 |
| Rectum AND TAHBSO AND total vaginectomy (n =11) | 9.17 (1.13 – 198.70), p = 0.066 | 4.12 (0.44 – 92.36), p = 0.26 | >1000 (NA), p = 0.99 | >1000 (NA), p = 0.99 | >1000 (NA), p = 1.00 |
| *Left pelvic sidewall vessels* |  |  |  |  |  |
| No left vessel component (n = 134) | - | - | - | - | - |
| Lymphadenectomy (n = 104) | 1.16 (0.65 – 2.04), p = 0.62 | 1.31 (0.70 – 2.45), p = 0.39 | 1.31 (0.43 – 3.94), p = 0.63 | 1.51 (0.53 – 4.45), p = 0.44 | >1000 (NA), p = 1.00 |
| Distal internal iliac artery (n = 17) | 6.53 (2.26 – 21.75), p = 0.001 | 2.27 (0.72 – 6.55), p = 0.14 | 2.42 (0.34 – 11.16), p = 0.30 | 9.90 (2.77 – 35.33), p <0.001 | >1000 (NA), p = 1.00 |
| Proximal internal iliac artery and vein (n = 63) | 1.57 (0.82 – 2.96), p = 0.17 | 1.54 (0.75 – 3.08), p = 0.23 | 2.64 (0.90 – 7.87), p = 0.07 | 0.59 (0.09 – 2.55), p = 0.53 | 1.00 (NA), p = 1.00 |
| External iliac artery or vein +/- internal iliac artery or vein (n = 9) | 1.36 (0.28 – 5.45), p = 0.67 | 2.08 (0.42 – 8.44), p = 0.32 | 0.00 (NA), p = 0.99 | 0.00 (NA), p = 0.99 | 1.00 (NA), p = 1.00 |
| *Right pelvic sidewall vessels* |  |  |  |  |  |
| No right vessel component (n = 163) | - | - | - | - | - |
| Lymphadenectomy (n = 96) | 1.51 (0.87 – 2.61), p = 0.14 | 1.54 (0.83 – 2.84), p = 0.17 | 2.03 (0.70 – 5.96), p = 0.19 | 1.39 (0.51 – 3.66), p = 0.50 | 1.00 (NA), p = 1.00 |
| Distal internal iliac artery (n = 16) | 2.88 (1.00 – 8.30), p = 0.05 | 3.59 (1.20 – 10.45), p = 0.019 | 1.49 (0.08 – 9.18), p = 0.72 | 2.19 (0.32 – 9.40), p = 0.34 | >1000 (NA), p = 1.00 |
| Proximal internal iliac artery and vein (n = 43) | 2.51 (1.25 – 5.03), p = 0.01 | 2.74 (1.30 – 5.71), p = 0.007 | 5.09 (1.72 – 15.44), p = 0.003 | 0.75 (0.11 – 2.96), 0.71 | 1000 (NA), p = 1.00 |
| External iliac artery or vein +/- internal iliac artery or vein (n = 9) | 0.82 (0.12 – 3.57), p = 0.81 | 0.58 (0.03 – 3.33), p = 0.58 | 0.00 (NA), p = 0.99 | 1.91 (0.10 – 12.04), p = 0.56 | 1.00 (NA), p = 1.00 |
| *Left pelvic sidewall nerves* |  |  |  |  |  |
| No left nerve component (n = 312) | - | - | - | - | - |
| Obturator nerve (n = 8) | 3.69 (0.89 – 18.30), p = 0.08 | 3.46 (0.80 – 14.96), p = 0.085 | 1.80 (0.09 – 10.72), p = 0.59 | 0.00 (NA), p = 0.99 | 0.00 (NA), p = 0.99 |
| Single nerve root (n = 3) | 1.11 (0.05 – 11.70), p = 0.93 | 1.73 (0.08 – 18.30), p = 0.657 | 0.00 (NA), p = 0.99 | 0.00 (NA), p = 0.99 | 0.00 (NA), p = 0.99 |
| Multiple nerve roots, S2 or below (n = 3) | 4.43 (0.42 – 96.06), p = 0.23 | 6.91 (0.65 – 150.08), p = 0.12 | 0.00 (NA), p = 0.99 | 6.59 (0.30 – 71.45), p = 0.13 | 0.00 (NA), p = 0.99 |
| Multiple nerve roots, S1 and below (n = 1) | 0.00 (NA), p = 0.99 | 0.00 (NA), p = 0.99 | 0.00 (NA), p = 0.99 | 0.00 (NA), p = 0.99 | 0.00 (NA), p = 0.99 |
| Complete sciatic nerve (n = 0) | - | - | - | - | - |
| *Right pelvic sidewall nerves* |  |  |  |  |  |
| No right nerve component (n = 310) | - | - | - | - | - |
| Obturator nerve (n = 7) | 0.88 (0.12 – 4.15), p = 0.88 | 1.37 (0.19 – 6.51), p = 0.71 | 0.00 (NA), p = 0.99 | 0.00 (NA), p = 0.99 | 0.00 (NA), p = 0.99 |
| Single nerve root (n = 2) | 2.20 (0.09 – 55.92), p = 0.58 | 0.00 (NA), p = 0.99 | 13.09 (0.51 – 338.68), p = 0.07 | 0.00 (NA), p = 0.99 | 0.00 (NA), p = 0.99 |
| Multiple nerve roots, S2 or below (n = 5) | 1.46 (0.19 – 8.97), p = 0.68 | 2.29 (0.30 – 14.05), p = 0.37 | 3.27 (0.16 – 23.33), p = 0.30 | 3.27 (0.16 – 23.33), p = 0.30 | 0.00 (NA), p = 0.99 |
| Multiple nerve roots, S1 and below (n = 0) | - | - | - | - | - |
| Complete sciatic nerve (n = 3) | >1000 (NA), p = 0.98 | >1000 (NA), p = 0.99 | 0.00 (NA), p = 0.99 | 0.00 (NA), p = 0.99 | 0.00 (NA), p = 0.99 |
| *Pelvic floor / muscles* |  |  |  |  |  |
| No pelvic floor / muscle component (n = 204) | - | - | - | - | - |
| Levator ani (n = 106) | 3.69 (2.22 – 6.19), p <0.001 | 1.37 (0.19 – 6.51), p = 0.71 | 8.24 (2.90 – 29.53), p <0.001 | 1.11 (0.43 – 2.68), p = 0.82 | 1.93 (0.08 – 49.22), p = 0.64 |
| Levator ani AND unilateral ischial spine (n = 14) | 14.58 (4.32 – 66.65), p <0.001 | 0.00 (NA), p = 0.99 | 27.78 (6.38 – 130.85), p <0.001 | 0.00 (NA), p = 0.99 | 0.00 (NA), p = 1.00 |
| Levator ani AND bilateral ischial spine (n = 2) | 3.98 (0.16 – 101.97), p = 0.33 | 2.29 (0.30 – 14.05), p = 0.37 | 0.00 (NA), p = 0.99 | 13.57 (0.52 – 355.76), p = 0.07 | 0.00 (NA), p = 1.00 |
| Iliacus, iliopsoas OR iliac crest (n = 1) | >1000 (NA), p = 0.99 | >1000 (NA), p = 0.99 | 0.00 (NA), p = 0.99 | 0.00 (NA), p = 0.99 | 0.00 (NA), p = 1.00 |
| *Complexity* |  |  |  |  |  |
| Conventional supralevator PE (n = 41) | - | - | - | - | - |
| High-complexity supralevator PE (n = 164) | 2.10 (0.83 – 6.44), p = 0.15 | 1.82 (0.66 – 6.45), p = 0.29 | >1000 (NA), p = 0.99 | 1.54 (0.40 – 10.15), p = 0.58 | >1000 (NA), p = 1.00 |
| Conventional infralevator PE (n = 29) | 5.08 (1.61 – 18.18), p = 0.008 | 4.16 (1.20 – 16.98), p 0.03 | >1000 (NA), p = 0.99 | 0.70 (0.03 – 7.62), p = 0.77 | 1.00 (NA), p = 1.00) |
| High-complexity infralevator PE (n = 93) | 8.74 (3.40 – 27.21), p <0.001 | 6.11 (2.22 – 21.67), p = 0.001 | >1000 (NA), p = 0.99 | 1.84 (0.44 – 12.54), p = 0.46 | >1000 (NA), p = 1.00 |
|  |  |  |  |  |  |
| **Methods of reconstruction** |  |  |  |  |  |
| No omentoplasty (n = 116) | - | - | - | - | - |
| Omentoplasty (n = 211) | 1.15 (0.71 – 1.89), p = 0.58 | 1.39 (0.81 – 2.45), p = 0.24 | 1.71 (0.69 – 4.83), p = 0.27 | 0.27 (0.10 – 0.63), p = 0.004 | 0.55 (0.02 – 13.93), p = 0.67 |
| *Perineal reconstruction* |  |  |  |  |  |
| Supralevator PE (n = 205) | - | - | - | - | - |
| Primary closure only (n = 2) | >1000 (NA), p = 0.98 | 5.61 (0.22 – 144.46), p = 0.23 | 50.25 (1.78 – 1441.91), p = 0.009 | 13.64 (0.52 – 357.63), p = 0.07 | 0.00 (NA), p = 1.00 |
| Biological mesh reconstruction (n = 81) | 4.18 (2.42 – 7.31), p <0.001 | 3.13 (1.73 – 5.69), p <0.001 | 10.50 (3.62 – 38.04), p <0.001 | 0.90 (0.28 – 2.44), p = 0.84 | 2.55 (0.10 – 65.0), p = 0.51 |
| Myocutaneous flap reconstruction (n = 24) | 2.77 (1.12 – 6.65), p = 0.023 | 3.37 (1.31 – 8.28), p = 0.009 | 4.57 (0.61 – 24.83), p = 0.09 | 0.59 (0.03 – 3.17), p = 0.62 | 0.00 (NA), p = 1.00 |
| Composite flap and mesh (n = 15) | 5.82 (1.99 – 18.24), p = 0.001 | 4.91 (1.62 – 14.66), p = 0.004 | 12.56 (2.27 – 63.63), p = 0.002 | 2.10 (0.31 – 8.65), p = 0.36 | 0.00 (NA), p = 1.00 |
| *Supralevator pelvic conduits* |  |  |  |  |  |
| Without neobladder (n = 203) | - | - | - | - | - |
| With neobladder (n = 2) | 0.00 (NA), p = 0.99 | 0.00 (NA), p = 0.99 | 0.00 (NA), p = 1.00 | 0.00 (NA), p = 0.99 | 0.00 (NA), p = 1.00 |
| Without intestinal continuity (n = 188) | - | - | - | - | - |
| With intestinal continuity (n = 17) | 0.82 (0.18 – 2.66), p = 0.76 | 0.00 (NA), p = 0.99 | 0.00 (NA), p = 1.00 | 3.45 (0.72 – 12.67), p = 0.08 | 0.00 (NA), p = 1.00 |

Table S3 – Logistical regression for all manifestations of empty pelvis syndrome (EPS) for all core descriptors. By doses of radiotherapy in Gray (Gy) compared to 0 Gy. Magnitude of resection for each aspect of the UK Pelvic Exenteration Lexicon, compared to no resection in the corresponding pelvic compartment, and for complexity summary scores compared to conventional supralevator pelvic exenteration. For method of reconstruction compared to no corresponding reconstruction. Note that no pelvic conduits were made in infralevator pelvic exenterations so only supralevator cases were included in this analysis (n = 205), intestinal continuity refers to any colorectal or coloanal anastomoses formed. NA = not calculable, EBRT = external beam radiotherapy, IOERT = intraoperative electron radiotherapy, PE = pelvic exenteration, TAHBSO = total abdominal hysterectomy with bilateral salpingo-oophrectomy, OR = odds ratio, IQR = interquartile range, CI = confidence interval.

| **EPS core descriptors** | Univariate logistical regression for any EPS manifestation, OR (CI) p-value, n=105 | Multivariable logistical regression for any EPS manifestation, OR (CI) p-value, n=105 |
| --- | --- | --- |
| **Radiotherapy induced damage**  (Per 1 Gy increase in dose of EBRT)* | 1.01 (1.00 – 1.02); p = 0.02 | 1.00 (0.99 – 1.01); p = 0.94 |
| **Magnitude of surgery** |  |  |
| *Summarised by complexity classification* |  |  |
| Conventional PE (n = 70) | - | - |
| High-complexity PE (n = 257) | 1.60 (0.89 – 3.01), p = 0.13 | 2.05 (1.06 – 4.18), p = 0.039 |
| *Surgical history* |  |  |
| No previous pelvic resections | - | - |
| Previous pelvic resections | 1.20 (0.74 – 1.95), p = 0.46 | 0.77 (0.44 – 1.34, p = 0.37) |
| *Gender* |  |  |
| Female | - | - |
| Male | 1.56 (0.98 – 2.50), p = 0.063 | 1.28 (0.74 – 2.21), p = 0.37 |
| **Methods of reconstruction** |  |  |
| No omentoplasty (n = 116) | - | - |
| Omentoplasty (n = 211) | 1.13 (0.70 – 1.86), p = 0.62 | 0.64 (0.34 – 1.20), p = 0.17 |
| *Perineal reconstruction* |  |  |
| Supralevator PE (n = 205) | - | - |
| Biological mesh reconstruction (n = 81) | 4.28 (2.47 – 7.50), p <0.001 | 5.33 (2.77 – 10.52), p <0.001 |
| Myocutaneous flap reconstruction (n = 24) | 2.84 (1.15 – 6.82), p = 0.02 | 3.52 (1.32 – 9.31), p = 0.011 |
| Composite flap and mesh (n = 15) | 5.96 (2.04 – 18.70), p = 0.001 | 6.59 (2.04 – 22.87), p = 0.002 |

Table S4 – Multivariable logistic regression of all empty pelvis syndrome (EPS) core descriptors for any EPS manifestation, defined as at least one of an infected pelvic collection, chronic perineal sinus, pelvic bowel obstruction, or enteroperineal fistula. Note convergence failure occurred when including all significant individual pelvic compartmental ordinal scales from the UK Pelvic Exenteration Network Lexicon in the model. However, the model successfully converged when these variables were summarised using overall complexity classifications, while also accounting for gender-specific resections and prior pelvic resection. PE = pelvic exenteration, EBRT = external beam radiotherapy, Gy = Gray. *One patient had missing EBRT dosing data and was excluded from the model.
